# Optimal Transmission Performance of Quadrature Wireless Resonators at 3 T

**DOI:** 10.1002/mrm.70610

**Published:** 2026-09-24

**Authors:** Leila Sharipova, Dmitrii Tikhonenko, Christophe Vilmen, David Bendahan, Stefan Enoch, Redha Abdeddaim, Alena Shchelokova, Marc Dubois

**Affiliations:** ^1^ School of Physics and Engineering ITMO University Saint Petersburg Russian Federation; ^2^ Aix‐Marseille Université, CNRS Centre de Résonance Magnétique Biologique et Médicale Marseille France; ^3^ Aix‐Marseille Université, CNRS Centrale Med, Institut Fresnel Marseille France

**Keywords:** birdcage coil, breast MRI, electromagnetic coupling, quadrature resonator, transmit efficiency, wireless coil

## Abstract

**Purpose:**

To demonstrate a method for optimizing the efficiency of wireless resonators using tailored excitation from a dual‐transmit birdcage coil (BC), and to present the design and experimental investigation of a quadrature resonator for 3 T breast MRI application.

**Methods:**

A double‐mode quadrature resonator was designed to enhance both orthogonal components of the $B_{1}$ field in a 3 T BC. Flip‐angle maps measurements were performed on phantom to validate the predicted transmit efficiency gains. Numerical simulations were performed on a human voxel model to demonstrate the ability to optimize the $B_{1}$ field and SAR metrics.

**Results:**

Experiments confirmed that each linear polarization was enhanced by the resonator, yielding a 4.5‐fold improvement in the transmit efficiency compared with the reference BC under conventional circularly polarized (CP) excitation. The optimal excitation was derived and validated experimentally, leading to an additional 2% increase in transmit efficiency. Simulations revealed that the resonator provided a 5.9‐fold (left breast) and 5.2‐fold (right breast) improvement in SAR efficiency under conventional CP excitation. Tailored excitations further increased SAR efficiency by 15% (left breast) and 31% (right breast), approaching a 7‐fold improvement compared to the reference BC.

**Conclusion:**

A double‐mode quadrature resonator was demonstrated for 3 T MRI. Combined with dual‐transmit BC, it enabled $B_{1}$ focusing and significantly improved transmit and SAR efficiency. Our analytical model allowed the derivation of tailored BC excitation to approach the theoretical performance limit of wireless resonators.

## 1 Introduction

Magnetic resonance imaging (MRI) has revolutionized breast cancer diagnostics by providing exceptional soft tissue contrast without the use of ionizing radiation [1, 2]. Conventional breast MRI protocols include dynamic contrast‐enhanced MRI based on T_1_‐weighted gradient echo sequence, T_2_‐weighted sequences requiring inversion pulses [3]. Diffusion weighted imaging has been investigated more recently [4]. While 3 T systems offer an improved signal‐to‐noise ratio (SNR), their widespread clinical implementation faces challenges such as radiofrequency (RF) magnetic field ($B_{1}$) inhomogeneity and increased power deposition in biological tissue (SAR) [5, 6]. Improving transmit efficiency at 3 T is highly desirable especially for sequences requiring refocusing pulses or RF saturation. Moreover, $B_{1}$ inhomogeneities arise primarily in the body at 3 T from RF wavelength shortening in tissues due to their high permittivity. The breast, particularly the peripheral regions near skin‐air interfaces, is notably affected resulting in local signal loss.

The targeted MRI concept, utilizing wireless resonant structures, has emerged as a promising approach to address some of these limitations [7, 8, 9, 10]. These passive devices operate through electromagnetic coupling with the scanner's whole‐body birdcage coil (BC), focusing the $B_{1}$ field within specific regions of interest (ROI) [11, 12]. This field focusing enabled substantial improvements in both transmit efficiency and receive sensitivity while maintaining compatibility with a wide range of clinical MR scanners without hardware modifications.

Recent developments in metamaterial‐inspired resonators have demonstrated significant potential for MRI applications, offering a promising alternative to conventional coils [13, 14, 15, 16, 17]. Various designs, such as metasolenoids or Helmholtz coils, have been proposed for breast MRI at 3 T and have shown notable RF field enhancements [18]. However, most existing metamaterial‐inspired resonators are only sensitive to a single linear component of the circularly polarized (CP) RF magnetic field generated by the body coil. This polarization mismatch results in approximately half of the input power being lost during transmission [19, 20].

Recent studies have demonstrated that such single‐mode resonators do not operate optimally when driven by a BC coil with conventional CP excitation and could benefit from linearly polarized (LP) excitation [17]. Although, SAR remained a roadblock, as most of the input power had to be channeled through a single BC port.

One solution relies on quadrature resonators capable of enhancing both linear components of the RF field simultaneously. Such a configuration has been reported using two nested orthogonal linear resonators each interacting with one field component [21]. While this approach can improve performance, resonance frequencies and electromagnetic coupling between the two separate structures must be carefully controlled. In this context, a single resonator capable of supporting two degenerate and orthogonal resonances would present an elegant and practical solution.

Recent studies showed the benefit of introducing a passive four‐legs BC nested in a 3 T whole body coil [22] or a passive 16‐rung Litz cage in a 1.5 T whole body coil [23]. These quadrature resonators were designed to be detuned during the transmission phase and to enhance the $B_{1}^{-}$ field only during signal reception. Indeed, the BC coil is a well‐known example of a resonator with two degenerate orthogonal transverse modes with identical efficiency [24]. However, its accessibility is not suitable for breast MRI applications. These would benefit from the combination of one axial and one transverse mode within the same resonator positioned vertically in the scanner. A design of such a resonator has been proposed for 1.5 T MRI [25]. However, as each resonance exhibits a different level of RF field enhancement, the conventional CP excitation would result in an unbalanced elliptical polarization within the ROI and ultimately lead to suboptimal performance. This discrepancy reflects a well‐established fundamental limit; whereby axial resonators are more efficient than transverse ones for a similar footprint [26].

This work aims to demonstrate and validate a methodology for maximizing transmit efficiency in 3 T breast MRI by using a quadrature metamaterial‐inspired resonator (metaQ) in conjunction with the RF shimming capabilities of a dual‐transmit 3 T BC. Our primary objective is to show theoretically and experimentally that the excitation of the BC can be systematically optimized to compensate for the polarization distortion introduced by the resonator. This calibration is crucial for re‐establishing a true CP mode and/or maximizing $B_{1}$ field within the metaQ, thereby approaching the theoretical limit of transmit efficiency enhancement. Furthermore, we evaluate the RF safety aspects through numerical SAR analysis using a human voxel model. We demonstrate that active shimming in dual transmit BC can be leveraged to maximize SAR efficiency for either left or right breast ROIs.

## 2 Theory

In MRI, the transmit efficiency is determined by the circularly polarized component $B_{1}^{+}$ co‐rotating with the nuclear precession, while the counter‐rotating component $B_{1}^{-}$ is primarily associated with signal reception. These components are related to the Cartesian magnetic field components $B_{x}$ and $B_{y}$ as shown in Equation (1) [27].

(1) $$B_{1}^{+}=\frac{B_{x}+iB_{y}}{2};B_{1}^{-}=\frac{{\left(B_{x}-iB_{y}\right)}^{*}}{2}$$

The BC is equipped with two input ports capable of generating both Cartesian components of the RF magnetic field. Consequently, quadrature excitation (equal amplitude and a 90° phase delay) provides a single rotating component of the RF magnetic field, namely $B_{1}^{+}$, thereby improving transmit efficiency. Any other combination of $B_{x}$ and $B_{y}$ amplitudes and/or phases offsets yields an elliptical polarization. In all cases, regardless of the amplitude of $B_{x}$ and $B_{y}$, it can be shown that the magnitude of the $B_{1}^{+}$ component is maximized when the two cartesian components are in exact phase quadrature [15].

Our objective is to describe the interaction of an arbitrary elliptical drive with a quadrature resonator. We assume that the resonator affects each Cartesian component by a complex coefficient $\alpha e^{i\varphi_{x}}$ and $\beta e^{i\varphi_{y}}$ for $B_{x}$ and $B_{y}$, respectively. The initial BC field is represented by a normalized vector $\left[B_{x},B_{y}\right]=\left[\mathrm{iA}e^{i\varphi_{0}},\sqrt{1-A^{2}}\right]$, where $i=\sqrt{-1}$, A is an arbitrary amplitude parameter in the interval [0,1] and $\varphi_{0}$ is an arbitrary phase delay. For example, the case with $A=1/\sqrt{2}$ and $\varphi_{0}=0$ corresponds to the CP excitation.

After the interaction with the resonator, we can express the magnetic field vector as $\left[B_{x},B_{y}\right]=\left[\mathrm{iA}{\alpha e}^{i\left(\varphi_{0}+\varphi_{x}\right)},\beta \sqrt{1-A^{2}}e^{i\varphi_{y}}\right]$. As discussed above, a 90° phase delay maximizes $B_{1}^{+}$ amplitude regardless of the relative amplitudes of $B_{x}$ and $B_{y}$. This condition can be enforced as a calibration step by setting the initial $\varphi_{0}$ term to $\varphi_{0}=\varphi_{y}-\varphi_{x}$. Under this condition, the magnetic field vector becomes $\left[B_{x},B_{y}\right]=\left[\mathrm{iA}{\alpha e}^{i\varphi_{y}},\beta \sqrt{1-A^{2}}e^{i\varphi_{y}}\right]$ and the amplitudes $B_{1}^{+}$ and $B_{1}^{-}$ can be expressed:

(2) $$\left|B_{1}^{+}\right|=\frac{1}{2}\left(A\alpha +\beta \sqrt{1-A^{2}}\right);\left|B_{1}^{-}\right|=\frac{1}{2}\left(A\alpha -\beta \sqrt{1-A^{2}}\right)$$

From Equation (2), two specific values of the input field amplitude A can be derived: (i) the value $A_{\max}^{+}$ that maximizes $\left|B_{1}^{+}\right|$, obtained by canceling the derivative of $\left|B_{1}^{+}\right|$ with respect to A and (ii) the value of $A_{0}^{-}$ that nulls $\left|B_{1}^{-}\right|$, the counter‐rotating component of the $B_{1}$ field, leading to a true CP mode. For strictly positive α and β, the solutions are given in Equation (3):

(3) $$A_{\max}^{+}={\left(\frac{\beta^{2}}{\alpha^{2}}+1\right)}^{-1/2};\;A_{0}^{-}={\left(\frac{\alpha^{2}}{\beta^{2}}+1\right)}^{-1/2}$$

As expected, for a resonator enhancing both components equally (α=β), these two specific points in Equation (3) coincide, such that the maximum of $\left|B_{1}^{+}\right|$ occurs simultaneously with the cancelation of $\left|B_{1}^{-}\right|$. However, for an unbalanced resonator (α≠β), after correction of phase perturbation, the maximum of $\left|B_{1}^{+}\right|$ shifts away from the zero of $\left|B_{1}^{-}\right|$, meaning that the maximum of $\left|B_{1}^{+}\right|$ corresponds to a specific elliptical excitation and that $\left|B_{1}^{-}\right|$ is not zero at this specific excitation.

These analytical results also confirm our previous observations reported in Reference [17] that in the limit of strongly asymmetric enhancement (α≪β) the maximum of $\left|B_{1}^{+}\right|$ is obtained for $A_{\max}^{+}=0$ corresponding to a pure linear excitation aligned with the resonator preferential component.

## 3 Methods

### 3.1 Coils' Design

The metaQ is based on an array of coupled split‐loop resonators (SLRs). As shown in Figure 1A, the design comprises eight SLRs interconnected by two vertical copper strips, all fabricated on a 2‐mm‐thick FR‐4 substrate. With overall dimensions of 165 × 165 × 162 mm^3^ selected to accommodate the average human breast, the SLR strips are each 3 mm wide (*w*) and arranged in two groups of four, with adjacent strips within a group spaced 8 mm apart (*d*) and separated by a central gap of 30 mm (*h*) between the groups. Each resonator features two symmetrical gaps (*g* = 3 mm) loaded with capacitors.

**FIGURE 1:**
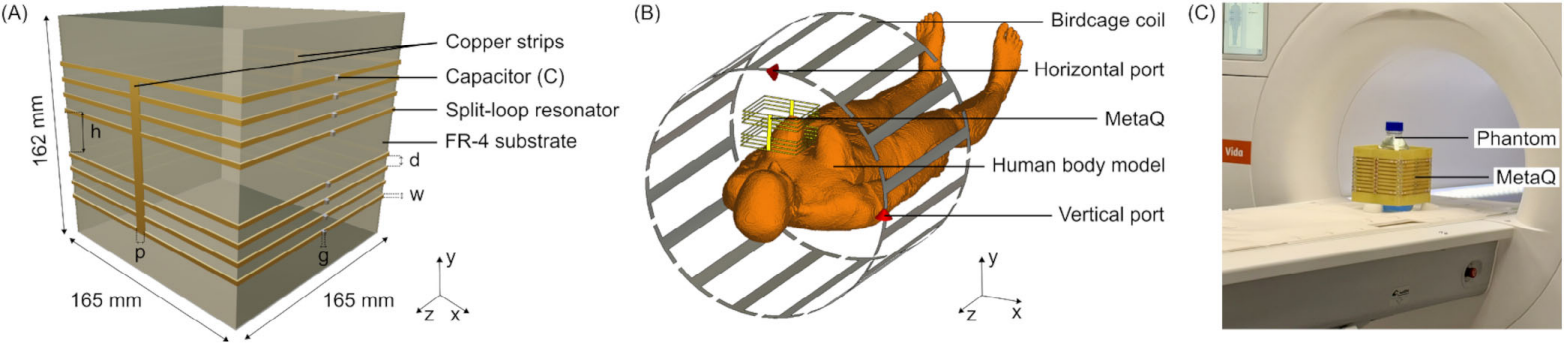
(A) Schematic view of the proposed metaQ structure design. (B) General view of the numerical simulation setup with the human voxel model and the 3 T BC coil. (С) Experimental setup showing the metaQ prototype placed around the homogeneous phantom inside the MRI scanner bore.

The operating principle relies on the resonant enhancement of two orthogonal magnetic field components. A *y*‐polarized RF magnetic field excites a solenoid‐type resonance with a uniform circulating current within each individual SLR, thereby generating an induced magnetic field along the normal axis. Conversely, an *x*‐polarized magnetic field couples to a saddle‐type resonance with longitudinal loops formed by the vertical strips and the SLR layers. Due to the symmetry of the current distribution, the normal component of the magnetic field undergoes destructive interference, and only the transverse magnetic field along the *x*‐axis is preserved.

### 3.2 MetaQ Manufacturing

The metaQ prototype was assembled from four etched FR‐4 substrates (165 × 165 × 2 mm^3^). All SLRs were interconnected at the corner with copper strips. The collective structure was tuned to the proton Larmor frequency of 123.2 MHz, corresponding to the 3 T MRI scanner used, by adjusting the capacitance values (*C* = 1.7 pF, Johanson Technology and Knowles) and the width of the connecting copper strips (*p* = 8 mm).

The resonant characteristics of the metaQ were evaluated using a vector network analyzer (VNA; Anritsu MS2026C). A 60‐mm‐diameter nonresonant pick‐up loop antenna, positioned above and on the side the metaQ, was used to measure the reflection coefficient ($S_{11}$ parameter). The VNA was calibrated with the coaxial cable and the pick‐up loop configured as a short circuit prior to measurements. Additionally, a dual‐loop sniffer probe was used to measure the Q factor of each resonance. Q‐factor was evaluated as the ratio of the loop resonant frequency to the bandwidth evaluated at −3 dB from the *S*_21_ maximum. An homogeneous cylindrical phantom (125 mm in diameter, 210 mm in height) with material properties (*ε* = 61.9, *σ* = 0.80 S/m; MVG body liquid 300) was used has load for resonator tuning and MR experiments.

### 3.3 Experimental Setup

All phantom experiments were conducted in a 3 T Siemens MAGNETOM Vida MRI scanner. The dual transmit interface of the scanner allows us to reliably set the amplitude and phase delay on each port of the BC, each corresponding to the vertical and horizontal linear polarizations.

Flip angle (FA) maps were acquired in the sagittal orientation using a preconditioning RF pulse followed by a turbo flash readout sequence [28] with the following parameters: TE/TR = 7030/1.86 ms, FA = 8°, Sinc pulse = 80°, acquisition matrix = 128 × 128, FOV = 289 × 289 mm, slice thickness = 10 mm and interslice gap = 20 mm.

For each experimental test, the reference voltage used by the scanner for RF power calibration was adjusted to reach a target value of 80° in the center of the phantom. This procedure ensured the accuracy of the FA mapping method [28]. The transmit efficiency was later quantified as the mean FA within a defined ROI divided by the corresponding input reference voltage.

Signal acquisitions were performed with a 3D Gradient echo sequence without acceleration, TR/TE = 5/2.3 ms, FA = 5°, acquisition matrix = 128 × 128 × 48 and FOV = 280 × 280 × 240 mm. The same gradient echo sequence was performed without RF input to acquire noise samples and compute SNR. An additional series of GRE acquisitions were performed with a flexible 12‐channel receive coil array wrapped around the phantom for comparison.

### 3.4 Electromagnetic Simulations

All electromagnetic simulations were performed using the finite element method in the time‐domain solver of CST Microwave Studio Suite 2022 (Dassault Systèmes). The simulation environment incorporated a whole‐body high‐pass quadrature BC. The 16‐leg BC was tuned and matched to 123.2 MHz using 12 pF capacitors (nominal resistive loss: 0.03 Ω). Its complete geometric parameters included a shield radius of 400 mm, a shield length of 850 mm, a resonant element radius of 350 mm, a resonant element length of 750 mm, and leg widths of 500 mm (Figure 1B). The coil was loaded with a multi‐tissue human voxel model based on the Ella model from the Virtual Family package [29], which was augmented with a breast model from the UWCEM Numerical Breast Phantom repository [30]. The model was positioned such that the isocenter of the coil was located at the chest wall, between the two breasts, to achieve a realistic and symmetric loading of the birdcage coil. In all simulations, the metaQ configuration utilized identical component values, specifically capacitors of 1.84 pF and connecting strip widths (*p*) of 11.5 mm.

The performance of the metaQ was evaluated through electromagnetic simulations by analyzing its transmit efficiency and SAR. Several configurations were considered, including the BC operating alone and the BC coupled with the metaQ. Each configuration was simulated under different excitation modes, including CP, vertical linear polarization (VLP), and horizontal linear polarization (HLP). Impedance matching of the BC was performed for configurations involving the metaQ.

The transmit efficiency was quantified by calculating the root‐mean‐square (RMS) amplitude of the transverse RF magnetic field ($\left|B_{1}^{+}\right|$) within a defined ROI in the breast tissue, normalized to 1 W of accepted power. The ROI was delineated by the breast tissue volume limited by the chest wall.

The local SAR was evaluated by averaging the spatial SAR distribution over 10 g of tissue (${\mathrm{SAR}}_{\mathrm{av}.10g}$). Distribution maps of ${\mathrm{SAR}}_{\mathrm{av}.10g}$ were constructed in the axial plane passing through the local SAR maximum. All SAR parameters were normalized to 1 W of total accepted power. The SAR efficiency was calculated as the ratio of the $\left|B_{1}^{+}\right|$ field in the ROI to the square root of the maximum local ${\mathrm{SAR}}_{\mathrm{av}.10g}$.

## 4 Results

### 4.1 Experimental Studies

Figure 2 presents the reflection coefficient ($S_{11}$) results for the metaQ resonator in its unloaded and phantom‐loaded conditions. In the unloaded state, the metaQ exhibited a sharp resonance at the Larmor frequency (123.2 MHz), regardless of whether the antenna was positioned above the structure (Figure 2A) or laterally (Figure 2B). As expected, loading with the phantom resulted in a reduction in the quality factor (seen as a broadening of the resonance), whereas the resonance frequency remained tuned to 123.2 MHz. The “saddle mode” resonance (horizontal polarization) presented a Q‐factor 160 unloaded and 40 loaded with our cylinder phantom. The “solenoid mode” resonance (vertical polarization) presented a Q‐factor of 87 unloaded and 20 in the loaded case.

**FIGURE 2:**
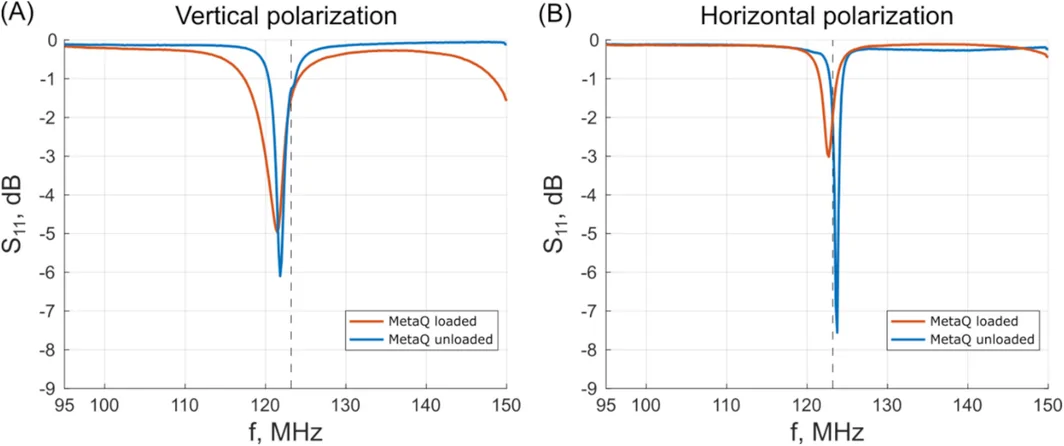
Reflection coefficient of the metaQ for (A) vertical and (B) horizontal polarizations. The blue curve represents the $S_{11}$ of the pickup loop antenna measured on the surface of the metamaterial. The red curve shows the $S_{11}$ when the metamaterial coil is loaded with a phantom. The vertical line denotes the working frequency of the 3 T MRI scanner at 123.2 MHz.

The experimentally measured FA maps demonstrate the performance of the BC under three distinct excitation polarizations, comparing the body coil alone (Figure 3A–C) to the system integrated with the metaQ (Figure 3D–F). Field profiles across the region of interest are presented in Figure 3G,H to illustrate the FA inhomogeneity in the presence of the metaQ. The resonator significantly reduced the reference voltage required to achieve the target FA across all excitation modes, thereby improving the transmit efficiency. Quantitative analysis revealed that the presence of the metaQ enhanced the transmit efficiency by factors of 5.27 for VLP, 3.53 for HLP, and 4.46 for CP. Although VLP showed the highest relative improvement, the CP mode yielded a 23% higher absolute transmit efficiency.

**FIGURE 3:**
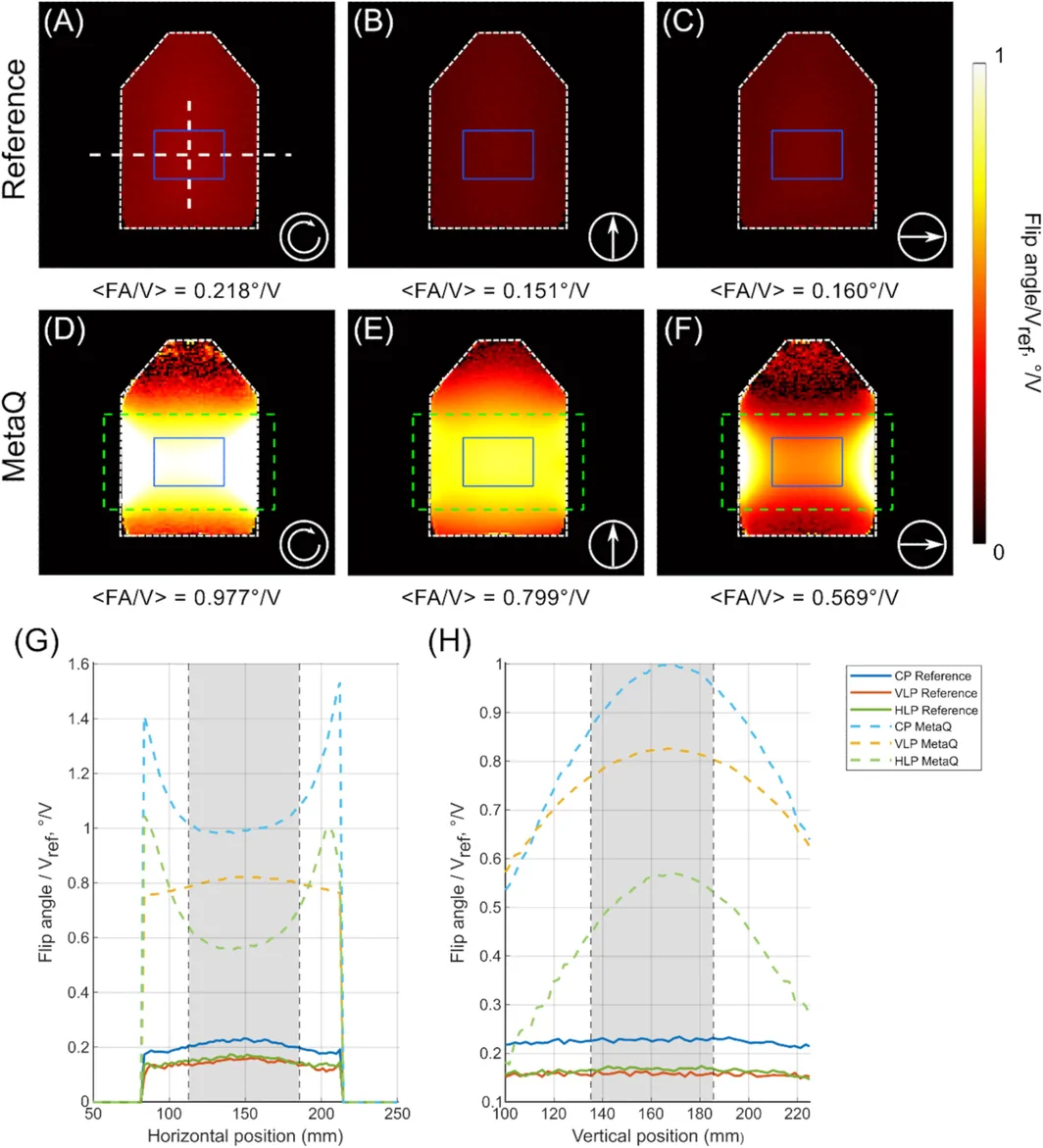
Flip‐angle maps acquired with the BC without (A–C) and with (D–F) the metaQ for polarization excitation modes (CP, HLP, VLP, as indicated on the maps). All FA maps are normalized by the reference voltage used for each acquisition. The dashed cross in (A) marks the position for the 1D profile extraction; the dashed contour in (D–F) outlines the metaQ. Panels (G) and (H) show the FA profiles extracted along the horizontal and vertical dashed lines, respectively; the gray‐shaded area indicates the ROI with its boundaries marked by vertical dashed lines.

Our next objective was to optimize the BC excitation to maximize the metaQ transmit efficiency. The first step consisted in correcting the additional phase delay introduced by the resonator. The transmit efficiency was plotted as a function of the phase delay between the two ports (Figure 4). A maximum transmit efficiency was observed at a phase delay of 75° (Figure 4A), indicating that the optimal quadrature phase delay was obtained between the two linear components.

**FIGURE 4:**
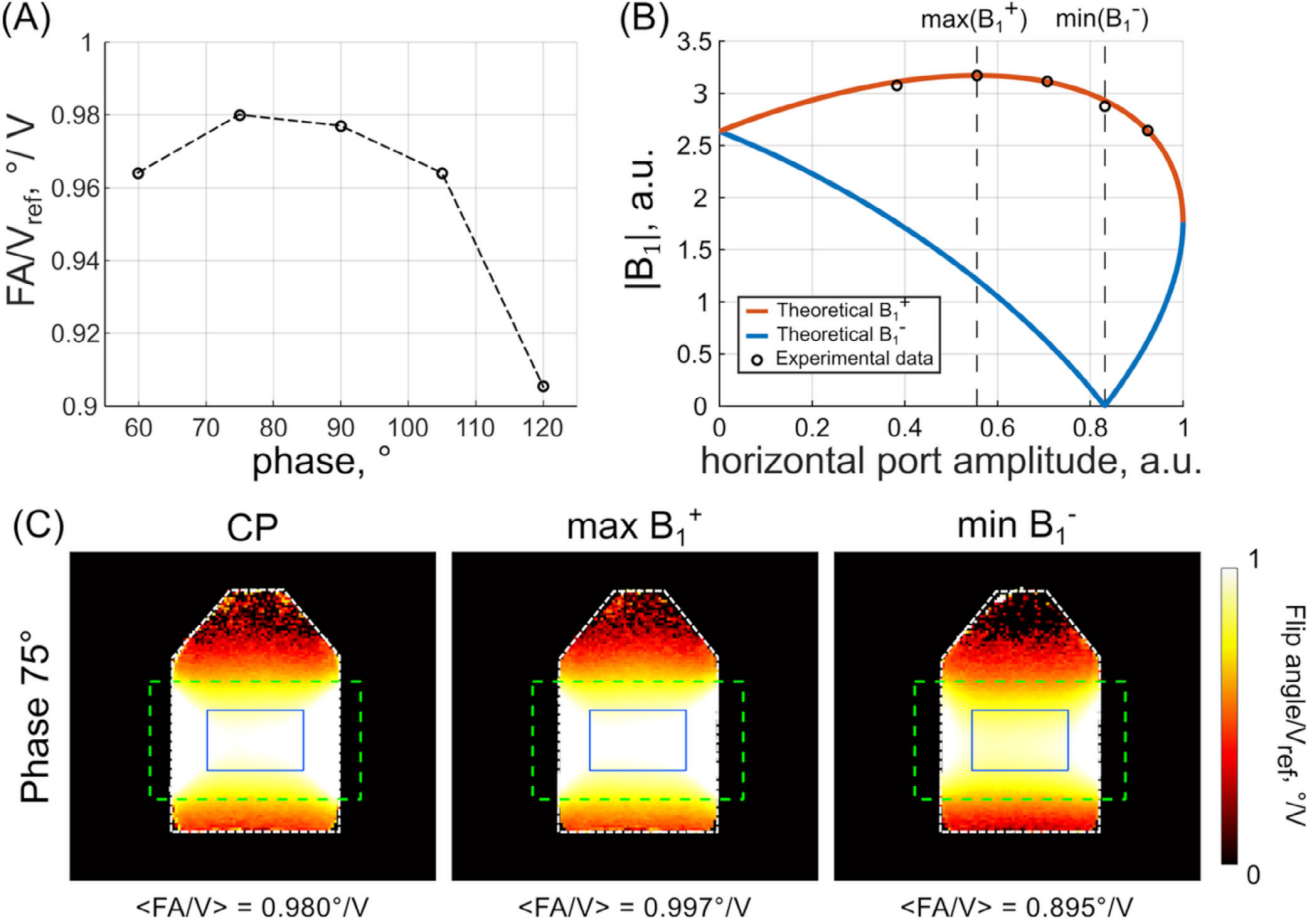
(A) $B_{1}$ efficiency (FA/V_ref_) as a function of the phase difference between BC ports, with input power equally distributed. (B) Theoretical |$B_{1}^{+}$| and |$B_{1}^{-}$| from type of excitation (red and blue curves respectively) with experimental data obtained during MR measurements (black dots). (C) Flip‐angle maps for the BC in CP mode and the metaQ, shown for a 75° excitation with different input power distributions across the ports.

As shown in Figure 3, the horizontal and vertical components of the magnetic field were enhanced by a factor α = 3.53 and 𝛽 = 5.27 respectively. Based on these values, the theoretical calculation of the $B_{1}^{+}$ and $B_{1}^{-}$ amplitude (solid lines) in the metaQ resonator obtained with Equation (2) with the amplitude $A_{x}$ swept from 0 to 1 is presented in Figure 4B. The amplitude $A_{\max}^{+}$ (maximum of $B_{1}^{+}$ amplitude) to be delivered to the horizontal port was evaluated with Equation (3), $A_{\max}^{+}=\left[B_{x}\right]=0.55$. Similarly, the amplitude $A_{0}^{-}$ (zero of $B_{1}^{-}$ amplitude) to be delivered to the horizontal channel was $A_{0}^{-}=\left[B_{x}\right]=0.84$.

These different excitations were tested experimentally with the dual‐transmit BC together with the balanced excitation and two more data points ($\left[B_{x}\right],\left[B_{y}\right]$) = (0.37, 0.92) and (0.92, 0.37) for a total of five different excitations shown with black circles in Figure 4B. Only the value for maximum $B_{1}^{+}$ amplitude was used to normalize the experimental transmit efficiency in FA/V to the arbitrary unit used in the theory. We observed that the transmit efficiency obtained from all experiments followed nicely the theoretical prediction. Figure 4C presented the normalized flip angle map for the balanced or CP excitation ($\left[B_{x}\right],\left[B_{y}\right]$) = (0.71, 0.71) as well as ($\left[B_{x}\right],\left[B_{y}\right]$) = (0.55, 0.84) and (0.84, 0.55). As expected, the second case, in the middle column, showed a transmit efficiency of 0.997 deg/V compared to 0.980 deg/V obtained with the phase corrected balanced excitation.

Figure 5 presents the SNR obtained with the metaQ. The performance of the metaQ resonator was compared to two reference cases with the BC alone and in presence of a receive array. For the reference BC alone under CP excitation, the average SNR within the phantom ROI was 95.6. When the metaQ was added and driven with the same CP excitation, V_ref_ decreased accordingly and the SNR rose to 444.6. This SNR gain of 4.65 closely matches the transmit efficiency gain of 4.46 reported in Figure 3. For the 12‐channel receive coil array, the scanner required *V*_ref_ = 310 V and achieved an average SNR of 558.6 in the ROI, 25% higher than the metaQ.

**FIGURE 5:**
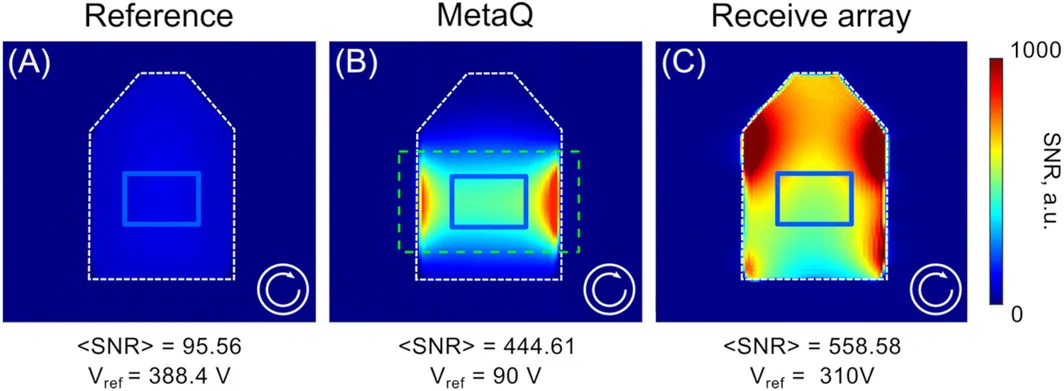
SNR maps for the reference BC alone (A), with metaQ (B) and with a receive array (C) for reception. In each case, the BC is transmitting with a CP excitation. The dashed line outlines the metaQ. The solid line marks the ROI.

### 4.2 Electromagnetic Simulations

Figure 6A–F presents simulated $\left|B_{1}^{+}\right|$ field distributions for the BC coil operating both without and with the metaQ positioned around the breast phantom under different excitation modes. Quantitative analysis revealed in the ROI_L_ a 7.8‐fold enhancement for CP, 8.3‐fold for VLP, and 7.6‐fold for HLP. Figure 6 (1–6) shows that metaQ integration substantially increased the ${\mathrm{SAR}}_{\mathrm{av}.10g}$ within the breast region. Notably, the ${\mathrm{SAR}}_{\mathrm{av}.10g}$ maximum shifted from the back muscles when using the BC alone to breast and abdominal areas with metaQ implementation. However, the overall SAR efficiency ($\left|B_{1}^{+}\right|.\mathrm{SA}R_{\mathrm{av}.10g}^{-0.5}$) demonstrated remarkable improvement, reaching 5.9‐fold for metaQ in the ROI_L_ under CP excitation compared to the reference BC alone.

**FIGURE 6:**
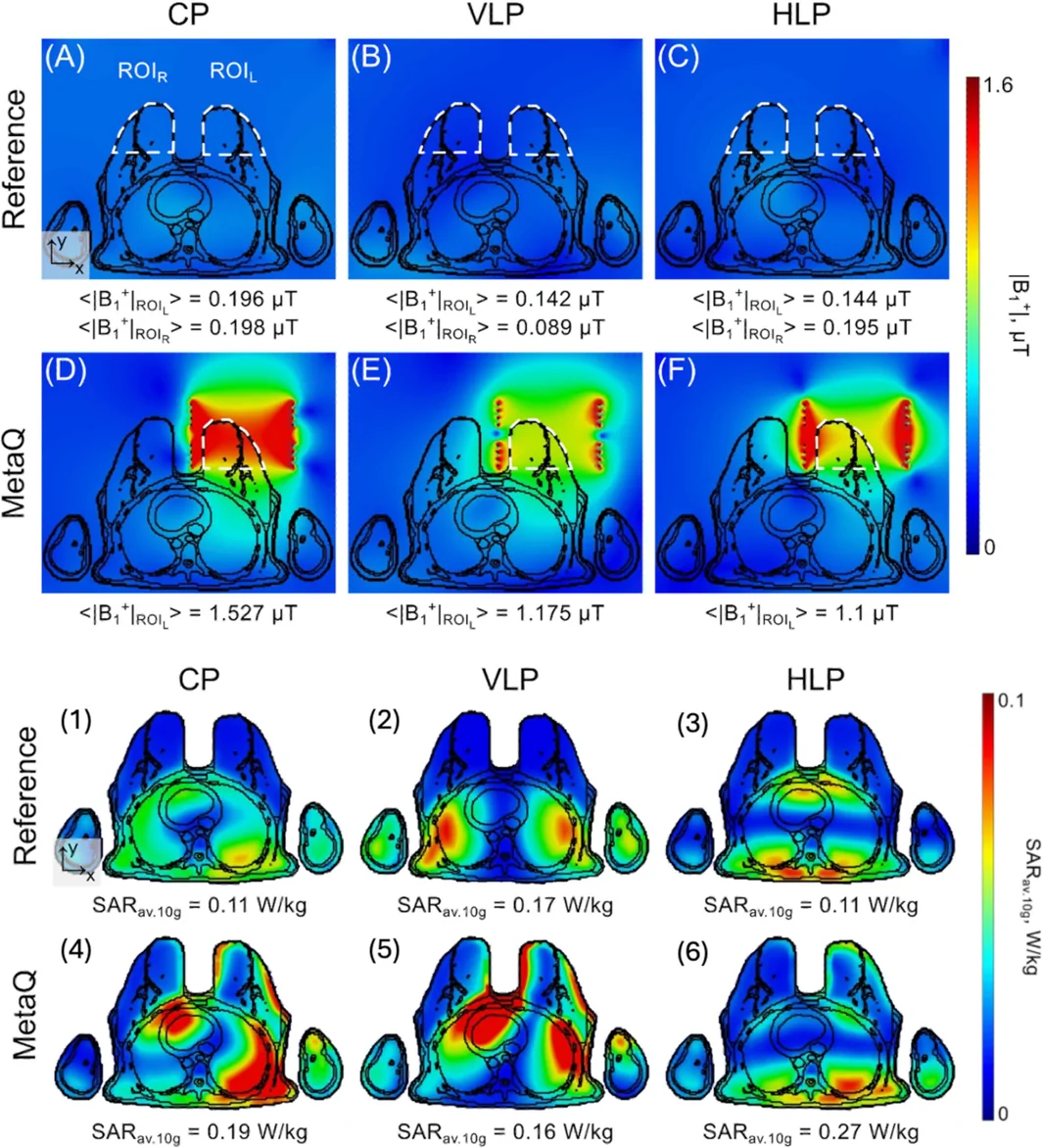
(A–F) Numerically calculated |$B_{1}^{+}$| maps in the central axial slice of a quadrature BC under different excitation modes, without (top row), with the metaQ for the left breast (lower row). The dashed line shows the region in which the amplitude of the |$B_{1}^{+}$| field distribution was calculated. (1–6) Numerically calculated SAR maps (averaged over 10 g of tissue) for a voxel model without (top row), with the metaQ for the left breast (bottom row) in the axial plane passing through the peak SAR value for a 1 W of total accepted power.

Our next objective was to investigate how the dual‐transmit BC could be used to adapt the BC excitation to the resonators in each position (left and right breast). The study on the right breast position brings similar conclusion and data is presented in Supporting Information. Unlike the experimental measurements where only the transmit efficiency was accessible, the numerical simulations allowed us to observe directly the impact of different BC excitations on the $\left|B_{1}^{+}\right|$, ${\mathrm{SAR}}_{\mathrm{av}.10g}$, and SAR efficiency.

Figure 7 shows the dependence of $\left|B_{1}^{+}\right|$, ${\mathrm{SAR}}_{\mathrm{av}.10g}$, and SAR efficiency in the ROI_L_ as a function of the phase delay between the two ports. The maximum $\left|B_{1}^{+}\right|$ amplitude was achieved with a phase delay of 125°, whereas the peak SAR efficiency occurred at 135° (Figure 7a–c). Leveraging the scanner's dual‐transmit capability, the power distribution across the BC ports was further optimized (Figure 7d–f). This revealed that the highest SAR efficiency was attained with input amplitude settings of *P*_1_ = 0.22 and *P*_2_ = 0.97 (Figure 7f). Consequently, the maximum SAR efficiency achieved for the left breast was 4.02 μT/√W/kg, representing a 15.2% increase compared to the convention CP excitation with equal power distribution (*P*_1_ = *P*_2_) and a 90° phase shift (Figure 7G–I).

**FIGURE 7:**
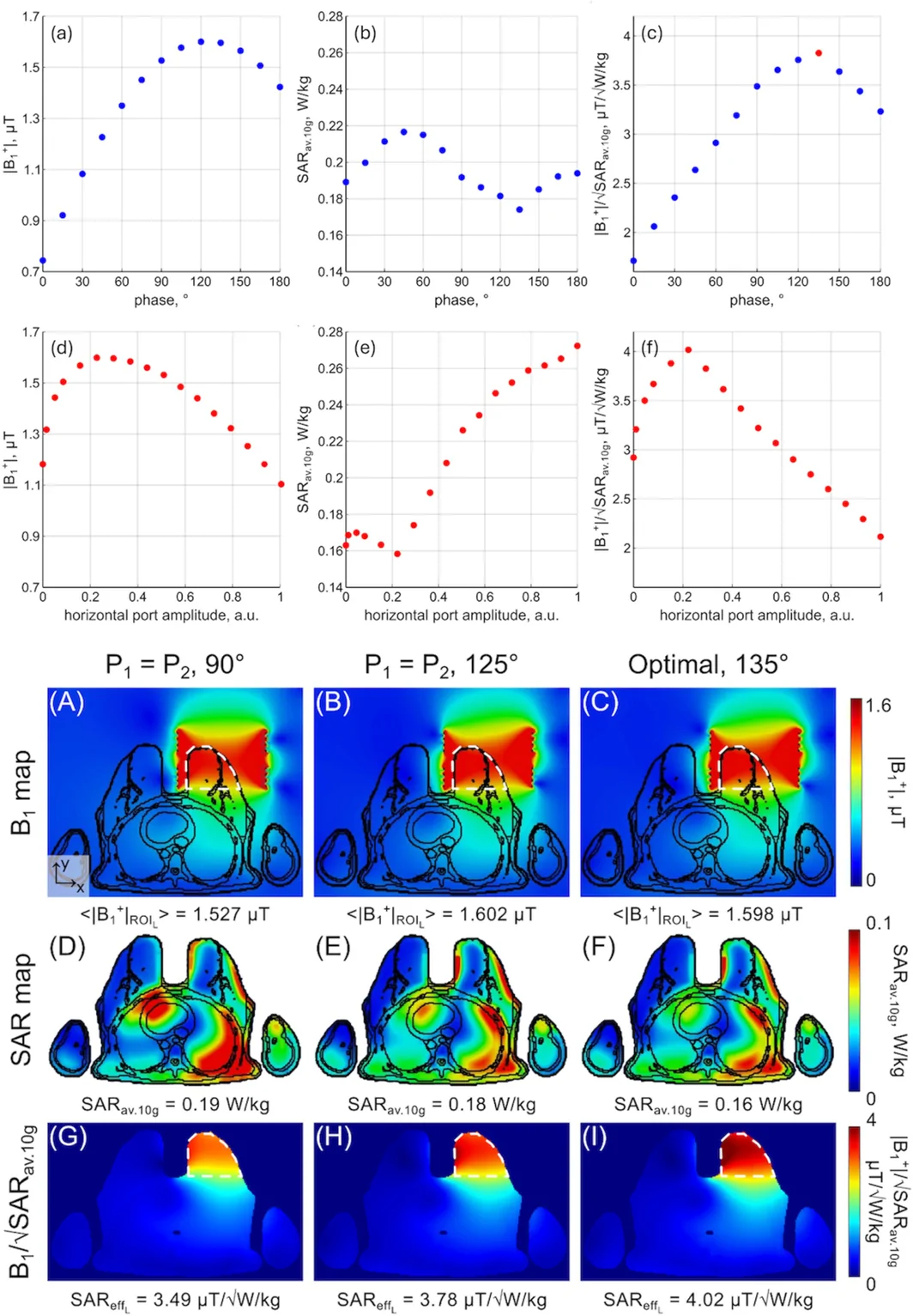
Simulated influence of RF excitation parameters on performance metrics for the left‐breast metaQ. (a–c) Dependence on the inter‐port phase delay (fixed amplitude) for (a) $\left|B_{1}^{+}\right|$, (b) ${\mathrm{SAR}}_{\mathrm{av}.10g}$, and (c) SAR efficiency. (d–f) Variation with the horizontal‐port amplitude (phase fixed at 135°) for the same three metrics. Associated field maps (A–C) |$B_{1}^{+}$|, (D–F) ${\mathrm{SAR}}_{\mathrm{av}.10g}$, and (G–I) SAR efficiency simulation result of metaQ for the left breast for different phase and input power distributions across the ports, from left to right: True CP, optimized phase, optimized amplitude & phase.

## 5 Discussion and Conclusions

Our metaQ resonator inspired from [25] integrates SLRs and copper strips into a single quadrature element. We demonstrated our ability to tune the two resonances of interest (solenoid and saddle modes) at the Larmor frequency by adjusting the positions and number of stacked SLRs, choosing the appropriate capacitance, and adjusting the width of the vertical strip connecting SLRs.

Experimentally, the CP excitation yielded the highest absolute transmit efficiency, validating the fact that the metaQ can significantly improve the transmit field in a target region. It was noted that the FA homogeneity across the phantom is reduced as the metaQ dimensions are smaller than the 2.5 L phantom used. In agreement with our theoretical prediction, we demonstrated that the optimization of phase delay and input power ratio between the BC ports increased the transmit efficiency by +2% from 0.977 to 0.997°/V. Despite a moderate increase, we wanted to insist on the generalization of our method and on the fact that a more unbalanced situation for the resonator and/or its loading could result in more striking improvement as we have seen in the numerical results on a more realistic model.

We found an SNR gain of 4.65 that closely matches the transmit efficiency gain of 4.55 obtained from the FA/V_ref_ ratio. This confirms that the metaQ used with the BC improves transmission and reception operations similarly. We compared the SNR obtained with the metaQ to a conventional setup in which there was a 12‐channel receive array. As expected, the receive array was able to outperform the passive resonator by more than 25% in SNR. Nonetheless, these acquisitions confirmed that the metaQ resonator did not introduce noise or artifact in GRE images.

A detailed analysis of SAR efficiency from these simulations reveals a performance asymmetry between the two orthogonal LP modes. For the metaQ, SAR efficiency was higher under VLP (2.94 μT/√W/kg) than under HLP (2.12 μT/√W/kg). This anisotropy indicates an intrinsic imbalance in the resonator's coupling to the BC's field components, likely stemming from its geometric design, its electromagnetic coupling to the BC ports, and electromagnetic interactions with the load. When using conventional CP excitation, the overall SAR efficiency for the combined BC and metaQ system demonstrated a remarkable 5.9‐fold improvement compared to the reference BC, indicating that the transmit efficiency increase outweighs the local SAR increase.

Interestingly, we identified a SAR efficiency discrepancy (3.49 and 3.14 μT/√W/kg) between left and right breasts ROIs despite using the same resonator, the same BC and the same conventional CP excitation. The data on the right breast is presented in the Supporting Information. One could wonder if this performance discrepancy is leading to a definitive loss of performance or whether the CP excitation is less efficient to drive the metaQ on the right breast. Different combinations of relative power and phase for the two transmit channels were tested to determine the optimal excitation for the metaQ. While the $\left|B_{1}^{+}\right|$ could be predicted with our theoretical method, it is not the case for the SAR which cannot be treated as a linear combination of single channel response. We observed that maximum $\left|B_{1}^{+}\right|$ would coincide with local minima of SAR for both ROIs. Consequently, the maximum SAR efficiency increased to 4.02 μT/√W/kg for metaQ in the ROI_L_ and 4.14 μT/√W/kg for metaQ in the ROI_R_. These represented the highest values achieved in this study with 6.7‐ and 6.9‐fold enhancement respectively compared to the reference BC and +15% and +31% improvement respectively when comparing the metaQ SAR efficiency with conventional CP excitation. We were able to demonstrate that the initial SAR efficiency discrepancy between left and right ROIs was corrected once the BC excitation was carefully optimized for each case. To our knowledge, there is no physical law guaranteeing the coincidence of maximum transmit efficiency and local minimum of SAR under varying BC excitation. Nonetheless, we present here a robust method that can be applied to other types of passive resonators.

While numerical SAR simulations indicate that the metaQ operates within safety limits under optimized excitations, direct temperature monitoring during RF transmission with fiber‐optic probes and long‐duration continuous RF protocols would be required to bring our study to in vivo validation. It is important to note the scope and limitations of the present work. Our study was limited to a proof‐of‐concept validation of the metaQ's tunability and field‐focusing properties in a well‐controlled phantom environment. The presented results establish a critical foundation for future research. A direct and necessary progression of this research is to perform human subject experiments, which are required to assess the coil's efficacy, patient‐specific adjustability, and practical value for breast MRI applications.

## Funding

This work was supported by Russian Federation project for Science and Universities (FSER‐2025‐0018); Amidex (AMX‐23‐CI‐01, AMX‐23‐CPJ‐10, AMX‐23‐EQ‐FO‐009, AMX‐19‐IET‐002).

## Conflicts of Interest

The authors declare no conflicts of interest.

## Supporting information


**Figure S1:** Numerically calculated $\left|B_{1}^{+}\right|$ maps in the central axial slice of a quadrature BC under different excitation modes, without (top row), with the metaQ for the left breast (middle row), and with the metaQ for the right breast (bottom row). The dashed line shows the region in which the amplitude of the $\left|B_{1}^{+}\right|$ field distribution was calculated.
**Figure S2:** Numerically calculated SAR maps (averaged over 10 g of tissue) for a voxel model without (top row), with the metaQ for the left breast (middle row), and with the metaQ for the right breast (bottom row) in the axial plane passing through the peak SAR value for a 1 W of total accepted power.
**Figure S3:** Simulated influence of RF excitation parameters on performance metrics for the right‐breast metaQ. (a–c) Dependence on the inter‐port phase delay (fixed amplitude) for (a) $\left|B_{1}^{+}\right|$, (b) ${\mathrm{SAR}}_{\mathrm{av}.10g}$, and (c) SAR efficiency. (d–f) Variation with the horizontal‐port amplitude (phase fixed at 135°) for the same three metrics. Associated field maps (A–C) $\left|B_{1}^{+}\right|$, (D–F) ${\mathrm{SAR}}_{\mathrm{av}.10g}$, and (G–I) SAR.
**Figure S4:** $\left|B_{1}^{+}\right|$ maps (top) and local SAR maps averaged over 10 g of tissue (bottom) for the active local transmit resonator in the left breast. Results are shown for CP, VLP, and HLP excitations (columns). Dashed line indicates the ROI used for $\left|B_{1}^{+}\right|$ quantification.

## Data Availability

The data that support the findings of this study are available from the corresponding author upon reasonable request.

## References

[1] G. L. Menezes , F. M. Knuttel , B. L. Stehouwer , R. M. Pijnappel , and M. van den Bosch , “Magnetic Resonance Imaging in Breast Cancer: A Literature Review and Future Perspectives,” World Journal of clinical oncology 5, no. 2 (2014): 61–70, 10.5306/wjco.v5.i2.61. PMC4014797 24829852

[2] R. M. Mann , N. Cho , and L. Moy , “Breast MRI: State of the Art,” Radiology 292, no. 3 (2019): 520–536, 10.1148/radiol.2019182947. 31361209

[3] W. B. DeMartini and H. Rahbar , “Breast Magnetic Resonance Imaging Technique at 1.5 T and 3 T: Requirements for Quality Imaging and American College of Radiology Accreditation,” Magnetic Resonance Imaging Clinics of North America 21, no. 3 (2013): 475–482, 10.1016/j.mric.2013.04.004. 23928238

[4] S. Bickelhaupt , F. B. Laun , J. Tesdorff , et al., “Fast and Noninvasive Characterization of Suspicious Lesions Detected at Breast Cancer X‐Ray Screening: Capability of Diffusion‐Weighted MR Imaging With MIPs,” Radiology 278, no. 3 (2016): 689–697, 10.1148/radiol.2015150425. 26418516

[5] H. Rahbar , S. C. Partridge , W. B. DeMartini , et al., “Clinical and Technical Considerations for High Quality Breast MRI at 3 Tesla,” Journal of Magnetic Resonance Imaging 37, no. 4 (2013): 778–790, 10.1002/jmri.23834. PMC6443571 23526757

[6] M. Dietzel , E. Wenkel , M. Hammon , et al., “Does Higher Field Strength Translate Into Better Diagnostic Accuracy? A Prospective Comparison of Breast MRI at 3 and 1.5 Tesla,” European Journal of Radiology 114 (2019): 51–56, 10.1016/j.ejrad.2019.02.033. 31005176

[7] A. Webb , A. Shchelokova , A. Slobozhanyuk , I. Zivkovic , and R. Schmidt , “Novel Materials in Magnetic Resonance Imaging: High Permittivity Ceramics, Metamaterials, Metasurfaces and Artificial Dielectrics,” Magma 35, no. 6 (2022): 875–894, 10.1007/s10334-022-01007-5. PMC9596558 35471464

[8] D. Darnell , T. K. Truong , and A. W. Song , “Recent Advances in Radio‐Frequency Coil Technologies: Flexible, Wireless, and Integrated Coil Arrays,” Journal of Magnetic Resonance Imaging 55, no. 4 (2022): 1026–1042, 10.1002/jmri.27865. PMC10494287 34324753

[9] Z. Guo , Y. Xu , S. Hu , et al., “Metamaterial‐Enhanced Magnetic Resonance Imaging: A Review,” Advanced Photonics Nexus 3, no. 5 (2024): 054001, 10.1117/1.APN.3.5.054001.

[10] A. Shchelokova , V. Ivanov , A. Mikhailovskaya , et al., “Ceramic Resonators for Targeted Clinical Magnetic Resonance Imaging of the Breast,” Nature Communications 11 (2020): 3840, 10.1038/s41467-020-17598-3. PMC7395080 32737293

[11] T. Sahara , S. Hashimoto , H. Tsutsui , et al., “Development of Inductively Coupled Wireless Radio Frequency Coil for Magnetic Resonance Scanners,” in Inaugural IEEE‐IES Digital EcoSystems and Technologies Conference (IEEE, 2007), 464–467, 10.1109/DEST.2007.372017.

[12] A. Alipour , A. C. Seifert , B. N. Delman , et al., “Improvement of Magnetic Resonance Imaging Using a Wireless Radiofrequency Resonator Array,” Scientific Reports 11, no. 1 (2021): 23034, 10.1038/s41598-021-02533-3. PMC8630230 34845314

[13] M. R. Smith , X. Zhai , K. N. Kurpad , R. D. Harter , and S. B. Fain , “Excite and Receive Solenoid Radiofrequency Coil for MRI‐Guided Breast Interventions,” Magnetic Resonance in Medicine 65, no. 6 (2011): 1799–1804, 10.1002/mrm.22759. 21590808

[14] V. Puchnin , G. Solomakha , A. Nikulin , A. W. Magill , A. Andreychenko , and A. Shchelokova , “Metamaterial Inspired Wireless Coil for Clinical Breast Imaging,” Journal of Magnetic Resonance 322 (2021): 106877, 10.1016/j.jmr.2020.106877. 33278812

[15] A. Jandaliyeva , V. Puchnin , A. Slobozhanyuk , and A. Shchelokova , “Control of the Near Magnetic Field Pattern Uniformity Inside Metamaterial‐Inspired Volumetric Resonators,” Photonics and Nanostructures – Fundamentals and Applications 48, no. 11 (2022): 100989, 10.1016/j.photonics.2021.100989.

[16] P. Tikhonov , A. Fedotov , G. Solomakha , and A. Hurshkainen , “A Wireless Bilateral Transceiver Coil Based on Volume Decoupled Resonators for a Clinical MR Mammography,” Journal of Magnetic Resonance 380 (2025): 107941, 10.1016/j.jmr.2025.107941. 40840018

[17] D. Tikhonenko , K. Rustomji , C. Vilmen , et al., “Optimal Excitation of Single Mode Resonators: Demonstration With a 3 T MRI Metasolenoid,” Journal of Magnetic Resonance 382 (2025): 107992, 10.1016/j.jmr.2025.107992. 41265244

[18] A. Jandaliyeva , V. Puchnin , and A. Shchelokova , “Volumetric Wireless Coils for Breast MRI: A Comparative Analysis of Metamaterial‐Inspired Coil, Helmholtz Coil, Ceramic Coil, and Solenoid,” Journal of Magnetic Resonance 359 (2024): 107627, 10.1016/j.jmr.2024.107627. 38280267

[19] G. H. Glover , C. E. Hayes , N. J. Pelc , et al., “Comparison of Linear and Circular Polarization for Magnetic Resonance Imaging,” Journal of Magnetic Resonance 64, no. 2 (1985): 255–270, 10.1016/0022-2364(85)90349-X.

[20] D. I. Hoult , C. N. Chen , and V. J. Sank , “Quadrature Detection in the Laboratory Frame,” Magnetic Resonance in Medicine 1, no. 3 (1984): 339–353, 10.1002/mrm.1910010305. 6571563

[21] V. Puchnin , A. Jandaliyeva , A. Hurshkainen , et al., “Quadrature Transceive Wireless Coil: Design Concept and Application for Bilateral Breast MRI at 1.5 T,” Magnetic Resonance in Medicine 89, no. 3 (2023): 1251–1264, 10.1002/mrm.29507. 36336799

[22] V. Zhurbenko , L. G. Hanson , W. Wang , and R. Jepsen , “Improving SNR of 3T MRI Using an Inductively Coupled Passive Birdcage,” in Proceedings of the International Society for Magnetic Resonance in Medicine, vol. 33 (Wiley, 2025), 4256.

[23] H. Zhu , M. L. Lang , Y. Yang , et al., “Detunable Wireless Litzcage Coil for Human Head MRI at 1.5 T,” NMR in Biomedicine 37, no. 3 (2024): e5068, 10.1002/nbm.5068. 37964107

[24] C. E. Hayes , W. A. Edelstein , J. F. Schenck , O. M. Mueller , and M. Eash , “An Efficient, Highly Homogeneous Radiofrequency Coil for Whole‐Body NMR Imaging at 1.5 T,” Journal of Magnetic Resonance 63, no. 3 (1985): 622–628, 10.1016/0022-2364(85)90257-4.

[25] Y. Jia , Y. Pang , R. Jin , et al., “A Unified Circular‐Polarization Metamaterial‐Inspired Resonator for Increasing SNR in Breast MRI,” Magnetic Resonance Imaging 121 (2025): 110403, 10.1016/j.mri.2025.110403. 40345562

[26] J. Mispelter and M. Lupu , “Homogeneous Resonators for Magnetic Resonance: A Review,” Comptes Rendus Chimie 11, no. 4–5 (2008): 340–355, 10.1016/j.crci.2007.10.003.

[27] M. V. Vaidya , C. M. Collins , D. K. Sodickson , R. Brown , G. C. Wiggins , and R. Lattanzi , “Dependence of B1+ and B1− Field Patterns of Surface Coils on the Electrical Properties of the Sample and the MR Operating Frequency,” Concepts in Magnetic Resonance Part B: Magnetic Resonance Engineering 46, no. 1 (2016): 25–40, 10.1002/cmr.b.21319. PMC5082994 27795697

[28] S. Chung , D. Kim , E. Breton , and L. Axel , “Rapid B1+ Mapping Using a Preconditioning RF Pulse With TurboFLASH Readout,” Magnetic Resonance in Medicine 64, no. 2 (2010): 439–446, 10.1002/mrm.22423. PMC2929762 20665788

[29] A. Christ , W. Kainz , E. G. Hahn , et al., “The Virtual Family—Development of Surface‐Based Anatomical Models of Two Adults and Two Children for Dosimetric Simulations,” Physics in Medicine and Biology 55, no. 2 (2010): N23–N38, 10.1088/0031-9155/55/2/N01. 20019402

[30] M. J. Burfeindt , T. J. Colgan , R. O. Mays , et al., “MRI‐Derived 3‐D‐Printed Breast Phantom for Microwave Breast Imaging Validation,” IEEE Antennas and Wireless Propagation Letters 11 (2012): 1610–1613, 10.1109/LAWP.2012.2236293. PMC4133125 25132808

