# Supplementary material for: Optimal Transmission Performance of Quadrature Wireless Resonators at 3 T
Source: Magn Reson Med. 2026 Sep 24;97(1):403–13. doi: 10.1002/mrm.70610 (PMC13746081; doi:10.1002/mrm.70610)
Supplement: Supplementary file 1 [file MRM-97-403-s001.pdf]

Supplemental Material

Title: Optimal transmission performance of quadrature wireless resonators at 3T

Authors: Leila Sharipova[1], Dmitrii Tikhonenko[2], Christophe Vilmen[2], David Bendahan[2], Stefan Enoch[3], Redha Abdeddaim[3], Alena Shchelokova[1], Marc Dubois[3]

[1] School of Physics and Engineering, ITMO University, Saint Petersburg, Russian Federation
[2] Centre de Résonance Magnétique Biologique et Médicale, Aix-Marseille Université, CNRS, Marseille, France
[3] Aix-Marseille Université, CNRS, Centrale Med, Institut Fresnel, Marseille, France

Table of content:

1-Numerical study of the metaQ resonator positioned on the right breast (page 1)

2-Numerical study of the metaQ resonator as an active coil without inductive coupling to the birdcage (page 5)

1- Numerical study of the metaQ resonator positioned on the right breast

Figure S1 presents simulated $|B_1^+|$ field distributions for the BC coil operating both without and with the metaQ positioned around the breast phantom under different excitation modes. The metaQ's interaction with both components of the BC's RF magnetic field resulted in superior transmit efficiency gains under CP compared to VLP and HLP.

Substantial field localization within the breast region was achieved with the metaQ. Quantitative analysis revealed in the ROI_L a 7.8-fold enhancement for CP, 8.3-fold for VLP, and 7.6-fold for HLP. Corresponding enhancements in the ROI_R reached 8.0-fold for CP, 17.3-fold for VLP, and 4.4-fold for HLP. Despite identical input power to BC channels, the resultant $|B_1^+|$ distribution exhibited significant asymmetry between ROIs. This effect stems from the complex electromagnetic interaction between the BC's dual-port excitation (90° phase shift) and the anatomical heterogeneity of the human body model.

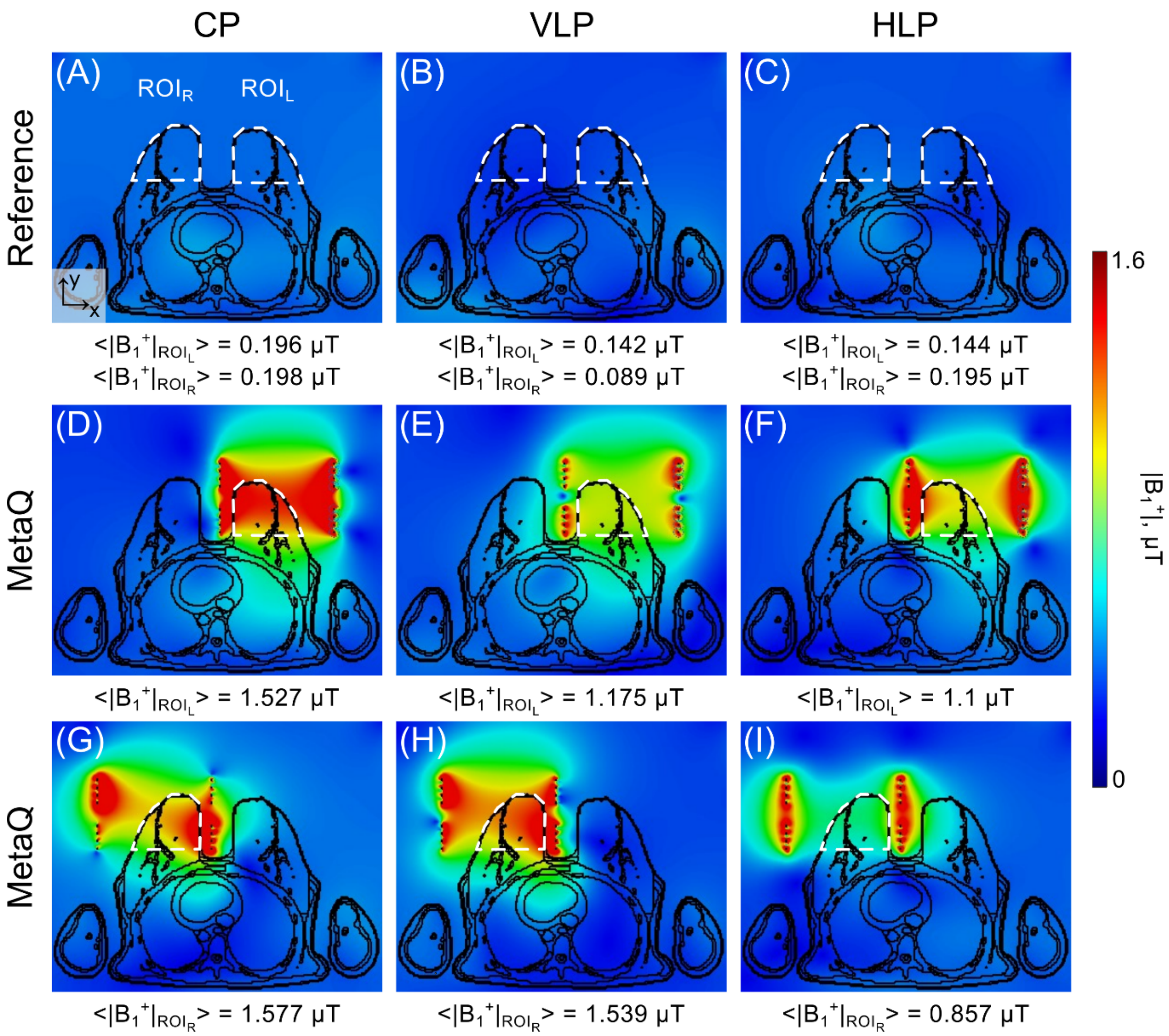

Figure S1. Numerically calculated $|B_1^+|$ maps in the central axial slice of a quadrature BC under different excitation modes, without (top row), with the metaQ for the left breast (middle row), and with the metaQ for the right breast (bottom row). The dashed line shows the region in which the amplitude of the $|B_1^+|$ field distribution was calculated

Figure S2 shows that metaQ integration substantially increased the $SAR_{av.10g}$ within the breast region. Notably, the $SAR_{av.10g}$ maximum shifted from the back muscles when using the BC alone to breast and abdominal areas with metaQ implementation. However, the metaQ's substantial improvement in transmit efficiency means that a significantly less input power is required to achieve the same $|B_1^+|$ field amplitude in the ROIs. Consequently, the overall SAR efficiency demonstrated remarkable improvement, reaching 5.9-fold for metaQ in the ROI_L and 5.2-fold for metaQ in the ROI_R under CP excitation compared to the reference BC alone.

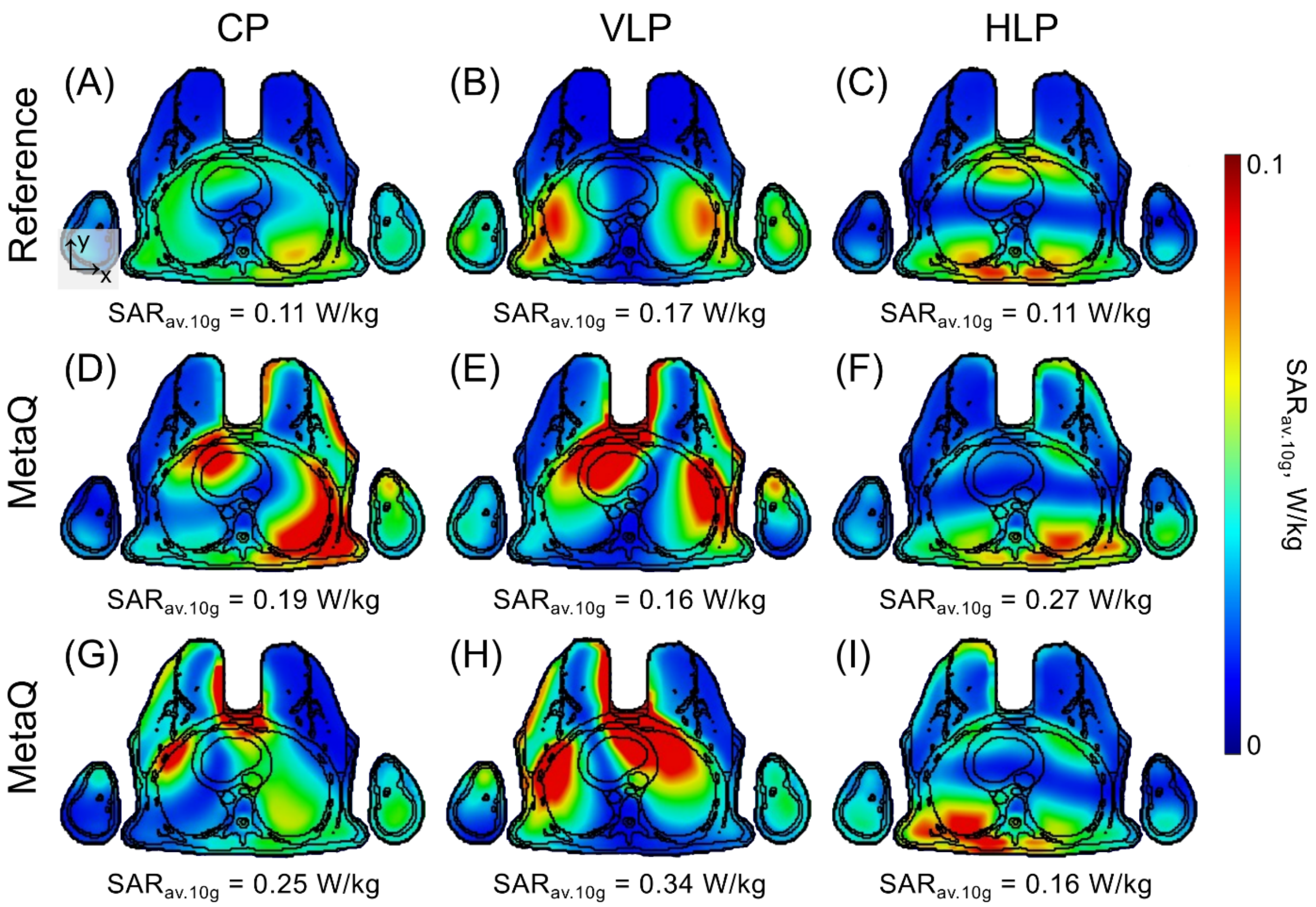

Figure S2. Numerically calculated SAR maps (averaged over 10g of tissue) for a voxel model without (top row), with the metaQ for the left breast (middle row), and with the metaQ for the right breast (bottom row) in the axial plane passing through the peak SAR value for a 1W of total accepted power

Figure S3 summarizes the simulation results for ROI_R, demonstrating how the $|B_1^+|$ field, $SAR_{av.10g}$, and SAR efficiency are influenced by the phase delay and input amplitude distribution between the BC ports. A similar optimization procedure was performed for the metaQ configuration in the ROI_R to determine its maximum achievable SAR efficiency. When varying the phase delay, the peak SAR efficiency was observed at 135° (figure S3c), whereas the maximum $|B_1^+|$ field amplitude occurred at 160°(figure S3a). Subsequent optimization, performed with the phase delay fixed at its optimal value of 135°, focused on the input power distribution across the ports (figure S3d-f). This optimization yielded a further improvement, achieving a maximum SAR efficiency of 4.14 μT/√W/kg at input amplitude settings of P_1 = 0.85 and P_2 = 0.53 (figure S3f). This final value represents a 32% increase compared to the baseline configuration with equal power distribution and a 90° phase shift as shown in the maps in figure S3G-I.

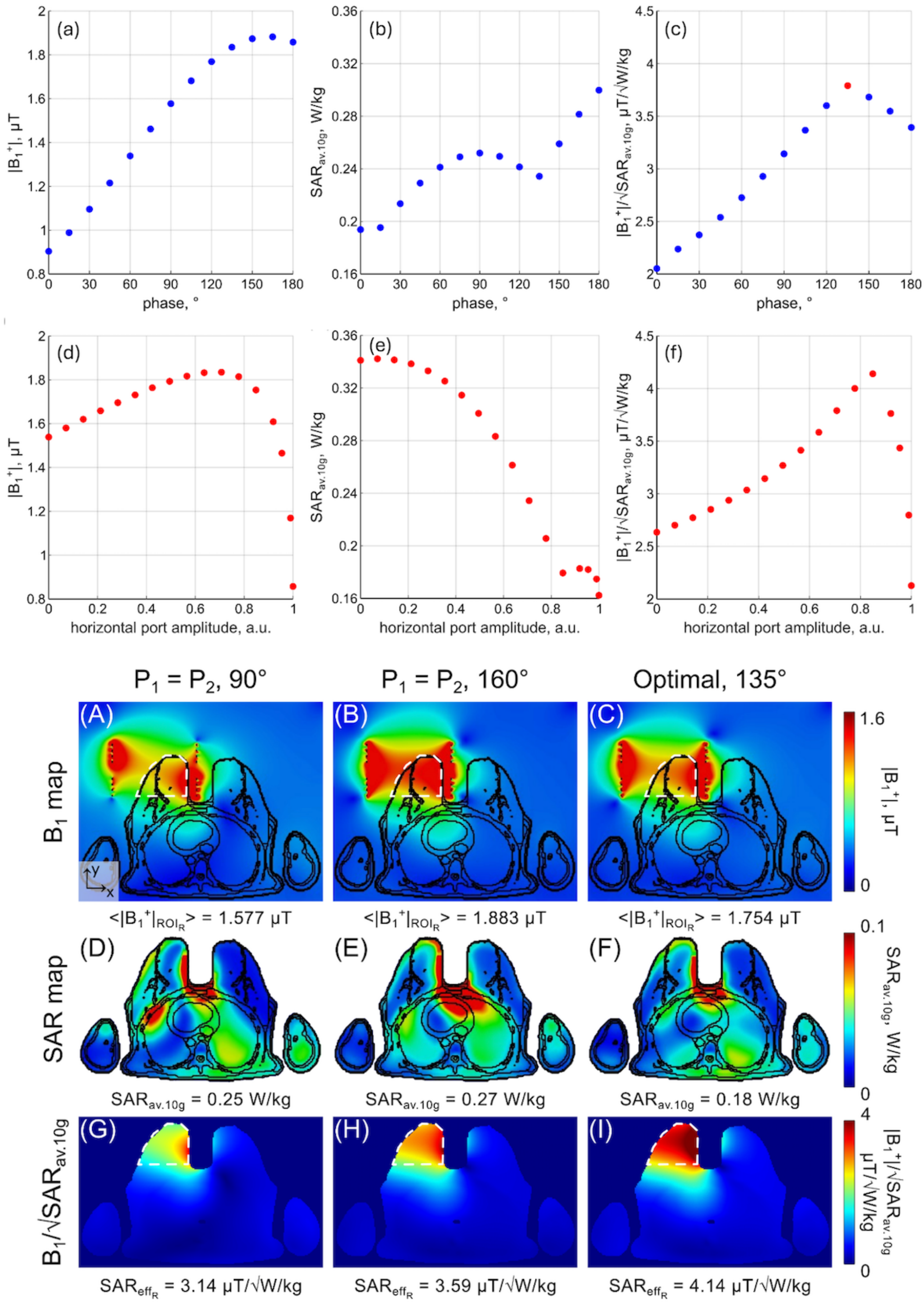

Figure S3. Simulated influence of RF excitation parameters on performance metrics for the right-breast metaQ. (a–c): Dependence on the inter-port phase delay (fixed amplitude) for (a) $|B_1^+|$, (b) $SAR_{av.10g}$, and (c) SAR efficiency. (d–f): Variation with the horizontal-port amplitude (phase fixed at 135°) for the same three metrics. Associated field maps (A-C) $|B_1^+|$, (D-F) $SAR_{av.10g}$ and (G-I) SAR efficiency simulation result of metaQ for the right breast for different phase and input power distributions across the ports, from left to right: true CP, optimized phase, optimized amplitude & phase.

2- Numerical study of the metaQ resonator as an active coil (without inductive coupling to the birdcage)

We performed a numerical comparison between metaQ and an active local transmit resonator of the same dimensions. The active local transmit resonator was modelled as a standalone transmit element driven directly by two independent RF ports matched to 50 Ω at the Larmor frequency of 123.2 MHz, with a 90° phase difference for circular polarization (CP). Three excitation polarizations were evaluated for the active resonator: CP, vertical linear (VLP), and horizontal linear (HLP). For the metaQ, we used the same polarizations, driven wirelessly via the whole-body birdcage coil under optimized excitation as described in the main manuscript. All simulations were performed on the same human voxel model (Ella, left breast ROI) using CST Microwave Studio. The numerical results of this comparison are presented in Figure S4. All reported $|B_1^+|$ and $SAR_{av.10g}$ values are normalized to 1 W of total accepted power.

For the active local transmit resonator under CP excitation, the $|B_1^+|$ in the left breast ROI was 1.627 μT. Under VLP and HLP, the values were 1.374 μT and 1.743 μT respectively, with HLP giving the highest field amplitude. For the metaQ under the same CP excitation, the $|B_1^+|$ was 1.527 μT. Thus, when both resonators were operating in CP mode, the active resonator delivered a slightly higher transmit field (about 6% higher) than the metaQ. Even under its most favorable polarization (HLP), the active resonator's advantage over the metaQ in CP mode was only about 14%.

However, the local SAR behavior shows an entirely different picture. For the active local transmit resonator under CP, the $SAR_{av.10g}$ was 0.94 W/kg, which is nearly five times higher than that of the metaQ under CP (0.19 W/kg). Under HLP, the active resonator's $SAR_{av.10g}$ reached 2.01 W/kg – more than ten times higher than the metaQ's CP value. This dramatic increase occurred because the active resonator concentrates all RF power directly into a compact structure placed against the breast, creating high current densities on the resonator legs and intense local power deposition. In contrast, the metaQ drews power wirelessly from the whole-body birdcage coil, which acts as a distributed source and a shield, spreading currents over a large surface and thereby reducing peak SAR. As a result, the SAR efficiency (defined for CP as $|B_1^+|/\sqrt{(SAR_{av.10g})}$) was much higher for the metaQ: 3.49 μT/√(W/kg) versus only 1.67 μT/√(W/kg) for the active resonator under CP. Even when the active resonator was operating under its best-performing HLP mode, its SAR efficiency dropped further to 1.23 μT/√(W/kg). Hence, for the same local SAR constraint, the metaQ could deliver more than twice the usable B_1^+ field of the active resonator.

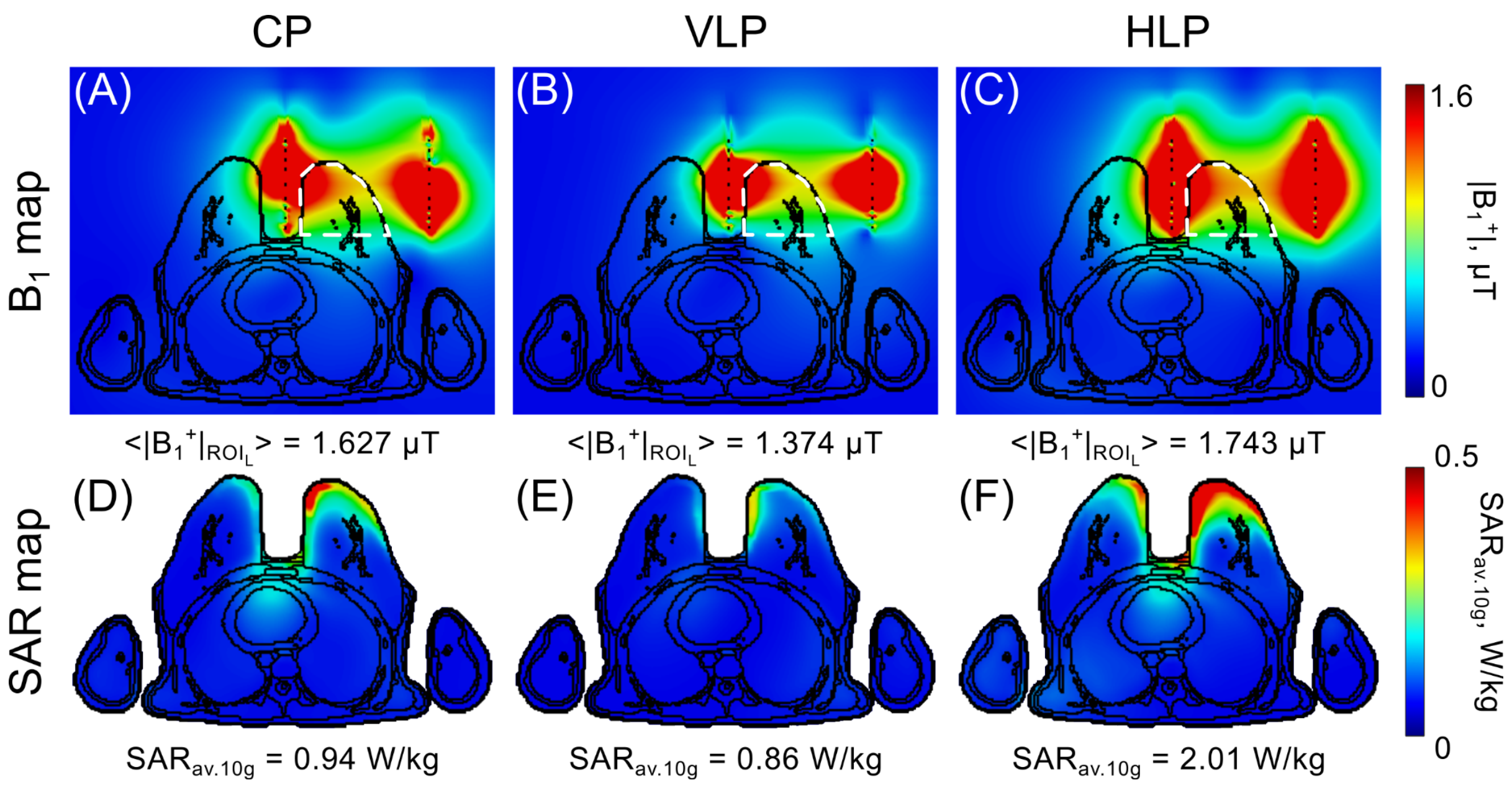

Figure S4. $|B_1^+|$ maps (top) and local SAR maps averaged over 10 g of tissue (bottom) for the active local transmit resonator in the left breast. Results are shown for CP, VLP and HLP excitations (columns). Dashed line indicates the ROI used for $|B_1^+|$ quantification

Breast MRI at 3T is often SAR-limited, especially in high-resolution or dynamic contrast-enhanced protocols. A higher SAR efficiency directly translates into either shorter acquisition times (while respecting the same SAR limits) or improved signal-to-noise ratio. The metaQ's superior SAR efficiency therefore offers a tangible clinical benefit. From a technical standpoint, the passive wireless approach requires no cables, no active decoupling circuits, and no scanner hardware modifications, making it immediately compatible with any 3T system equipped with a dual-transmit birdcage coil. The active local transmit resonator, in contrast, would need dedicated receive-only arrays, active detuning, and additional RF channels, substantially increasing system complexity and cost.

In conclusion, although an active local transmit resonator can produce slightly higher $|B_1^+|$, its much larger local SAR leads to a substantially lower SAR efficiency compared to the passive metaQ (1.67 vs 3.49 μT/√(W/kg) under CP). The wireless metaQ thus provides a superior trade-off between field enhancement and tissue heating, combined with practical advantages in simplicity and scanner independence.
